# Supplementary material for: C1q Is Recognized as a Soluble Autoantigen by Anti-C1q Antibodies of Patients with Systemic Lupus Erythematosus
Source: Antibodies (Basel). 2025 Nov 5;14(4):94. doi: 10.3390/antib14040094 (PMC12641849; doi:10.3390/antib14040094)
Supplement: Supplementary file 1 [file antibodies-14-00094-s001.zip › antibodies-3908099-supplementary.pdf]

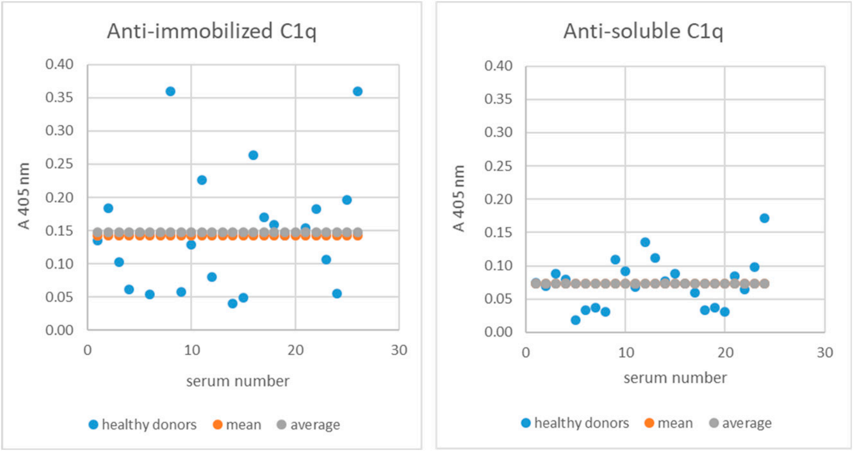

Supplementary Figure S1. **Presence of autoantibodies to immobilized or soluble C1q in sera of healthy donors.** Autoantibodies to immobilized C1q or soluble C1q in sera of healthy donors. 1 dot = 1 individual serum (n = 24).
